# Clinical High-Resolution Imaging of the Inner Ear by Using Magnetic Resonance Imaging (MRI) and Cone Beam Computed Tomography (CBCT)

**DOI:** 10.3390/jpm14060637

**Published:** 2024-06-14

**Authors:** Tomislav Santek, Erich Hofmann, Christian Milewski, Konrad Schwager, Andreas Prescher

**Affiliations:** 1 Department of Otorhinolaryngology Head and Neck Surgery, Klinikum Fulda gAG, 36043 Fulda, Germany; konrad.schwager@klinikum-fulda.de; 2 Department of Neuroradiology, Klinikum Fulda gAG, 36043 Fulda, Germany; ursi.hofmann@web.de; 3 HNO-Privatpraxis an der Börse Frankfurt, 60313 Frankfurt am Main, Germany; c.milewski@t-online.de; 4 Institute of Molecular and Cellular Anatomy-Prosektur, University Hospital RWTH Aachen, 52074 Aachen, Germany; aprescher@ukaachen.de

**Keywords:** truncation artifact, inner ear anatomy, visualization of inner ear, magnetic resonance imaging, cone beam computed tomography

## Abstract

Purpose: Imaging of the delicate inner ear morphology has become more and more precise owing to the rapid progress in magnetic resonance imaging (MRI). However, in clinical practice, the interpretation of imaging findings is hampered by a limited knowledge of anatomical details which are frequently obscured by artifacts. Corresponding review articles are as rare in journals as they are in reference books. This shortness prompted us to perform a direct comparison of imaging with anatomical whole-mount sections as a reference. It was the intention of this paper to compare the microscopic anatomy of a human inner ear as shown on anatomical whole-mount sections with high-resolution MRI and cone beam computed tomography (CBCT). Both are available in clinical routine and depict the structures with maximum spatial resolution. It was also a goal of this work to clarify if structures that were observed on MRI in a regular manner correlate with factual inner ear anatomy or correspond with artifacts typical for imaging. Methods: A fresh human anatomical specimen was examined on a clinical 3-Tesla MRI scanner using a dedicated surface coil. The same specimen was then studied with CBCT. In each imaging modality, high-resolution 3D data sets which enabled multiplanar reformatting were created. In the second step, anatomical whole-mount sections of the specimen were cut and stained. This process enabled a direct comparison of imaging with anatomical conditions. Results: Clinical MRI was able to depict the inner ear with remarkable anatomical precision. Strongly T2-weighted imaging protocols are exquisitely capable of showing the fluid-filled components of the inner ear. The macular organs, ampullar crests and cochlear aqueduct were clearly visible. Truncation artifacts are prone to be confused with the delicate membrane separating the endolymphatic from the perilymphatic compartment. However, it was not possible to directly depict this borderline. Conclusions: With the maximum resolution of magnetic resonance tomography, commonly used in everyday clinical practice, even the smallest details of the inner ear structures can be reliably displayed. However, it is important to distinguish between truncation artifacts and true anatomical structures. Therefore, this study can be useful as a reference for image analysis.

## 1. Introduction

Owing to the complex anatomy and physiology of the inner ear, diagnostics are based on a variety of tests.

Despite abundant available clinical and electrophysiological testing of the inner ear, a definite diagnosis of underlying pathologies is often made possible only by targeted imaging.

In previous decades, cross-sectional imaging modalities such as computed tomography (CT), MRI and CBCT have made their mark. The fast technical development of hardware and software has enabled an ever-increasing spatial resolution for the presentation of even tiny anatomical details. None of the modalities can claim to be superior. Rather, each imaging method has a complementary role. CT and CBCT are superior in the depiction of osseous structures such as the osseous labyrinth, whereas MRI is better able to present the fluid-filled compartments and the inner ear as well as the inner auditory meatus [1,2]. 

CBCT is a digital imaging technique using a three-dimensional conical X-ray bundle in combination with a flat panel detector. It distinguishes itself by its high sharpness of detail and superior spatial resolution. Based on several hundred projections, special mathematical procedures (back projection) create submillimeter slices which compose a 3D data set. Based on this slab which corresponds with the volume of interest, multiplanar secondary image reformatting can be performed at the examiner’s discretion.

MRI supplements high-resolution CT and CBCT in depicting the fluid-filled compartments of the inner ear without exposing the patient to the risk of ionizing radiation. T2-weighted sequences are especially suitable as they show a high inherent natural contrast between the bright endo- and perilymph on the one hand and the signal void of the surrounding bone and aerated spaces on the other. Using fast spin echo (FSE) techniques, a low slice thickness could be combined with a high in-plane spatial resolution [3]. It was 3D Fourier-transformed gradient echo sequences that made a slice thickness as low as 1 mm possible, enabling a more detailed delineation of the membranous labyrinth [4,5]. A true breakthrough came with the introduction of the 3D constructive interference in steady state (CISS) sequence which was introduced in the detailed analysis of the inner ear structures [6]. In fact, this sequence is specific for one manufacturing company; however, measuring protocols with similar imaging characteristics were introduced into the market by other companies.

X-ray-based images as in CT and CBCT can be degraded by beam hardening artifacts which usually can be neglected in clinical practice due to the wide window setting in routine temporal bone imaging. On the other hand, MRI of the inner ear has inherent shortcomings due to a bunch of imaging artifacts. Identifying artifacts and distinguishing them from factual anatomy can be difficult and has been addressed in previous work, most of which was conducted on scans of patients or volunteers using clinical hardware and software [7,8]. Therefore, it was the goal of our study to apply our best available imaging protocols on a temporal bone specimen and to perform a one-by-one comparison of slices from imaging scans with histologic sections of the same specimen. A secondary goal was to correctly denominate true anatomical structures and to create a reliable anatomical reference for clinical practice.

## 2. Materials and Methods

### 2.1. Temporal Bone Specimen

We examined a left-side fresh human temporal bone specimen of a 78-year-old male body donor of the body donation program of the RWTH Aachen. In order to minimize postmortal degradation of the delicate inner ear structures, the specimen was taken from the donor within a postmortem period of less than 24 h. There was no known disease of the ear and no signs of congenital defects or other abnormalities. MRI was performed within 24 h postmortem with the refrigerated fresh specimen. After fixation in 4% methanol-stabilized buffered formalin with a fixation duration of 14 days, CBCT was carried out. As a last step, the anatomical preparation of the temporal bone specimen was performed.

### 2.2. Imaging Protocols

#### 2.2.1. MRI

The examination was performed on a clinical scanner with a magnetic flux density (“field strength”) of 3 Tesla (Philips Ingenia, software version 5.4.1, Philips Healthcare, Best, the Netherlands). The specimen was enclosed in a cylindric plastic container (diameter 9 cm, height 5 cm) and was sealed off with an airtight screw cap. It was embedded in a physiologic 0.9% saline solution free from air bubbles. For the scan, the container was positioned in the center of a dedicated surface coil (dStream Knee 16 coil, Philips Healthcare, Best, The Netherlands). Apart from the usually performed planning scans, the examination protocol consisted of a heavily T2-weighted 3D-driven equilibrium (DRIVE) fast spinecho sequence with an echo time (TE) of 600 ms, a repetition time (TR) of 1287 ms and a turbo factor of 29.3. The reconstruction matrix was 960, with a scan percentage of 100% and a total duration time of roughly 43 min. This protocol resulted in extreme T2 weighting and provided high spatial resolution: the field-of-view (FOV) was 60 mm (*X* axis, left–right) × 60 mm (*Y* axis, anteroposterior) × 25 mm (*Z* axis, caudocranial). The reconstructed voxel size was 0.0625 mm × 0.0625 mm × 0.10 mm (*X*, *Y*, *Z*).

#### 2.2.2. Cone Beam CT

Imaging was performed on a clinical scanner (J. Morita MFG Corp., Kyoto, Japan, software version 2.630.370.3688). The FOV was 79.84 mm (*X*) × 79.84 mm (*Y*) × 80.32 mm (*Z*), with a voxel size of 0.16 mm (*X*, *Y*, *Z*). The exposure time was 17.5 s, with a voltage of 90 kV with 10 mA, a slice interval of 0.48 mm and a slice thickness of 0.960 mm.

#### 2.2.3. Anatomical Whole-Mount Sections

The temporal bone was fixated for 48 h in a 4% buffered (pH 6.9) formalin solution (article no. 1.00496.5000, Merck KGaA, Darmstadt, Germany). Subsequently, the labyrinth block was trimmed using a diamond band saw (Exakt Apparatebau GmbH & Co. KG, Norderstedt, Germany) to form a cube with an edge length of approximately 10 mm. The specimen was then processed according to the cutting–grinding technique described by Karl Donath [9]. 

In order to prepare the specimen for the cutting–grinding technique, it was embedded in methylmethacrylate (Technovit 9100, Heraeus-Kulzer GmbH, Hanau, Germany), according to the manual “Technovit Histotechnik” edited by the company. Finally, the completed slides were stained with toluidine blue–pyronine (ready-for-use toluidine blue–pyronin solution, Morphisto GmbH, Frankfurt/Main, Germany).

#### 2.2.4. Image Processing

Both 3D data sets of MRI as well as CBCT were archived in a clinical picture archiving and communication system (PACS) (Agfa Impax, version 6.5.2.1502, Agfa Healthcare GmbH, Bonn, Germany). Multiplanar reformatting was performed using the proprietary postprocessing module. The reformatted slice thickness was one pixel. With the aid of the histological whole-mount sections, we reformatted the analogous imaging plane to obtain a close match of imaging and histology. In the second step, we performed multiplanar reformatting in the three clinical standard planes (axial, coronal, sagittal). The implemented reference plane tool using a crosshair facilitated the correct assignment of anatomical structures in each of the three planes.

On the CBCT scans, a good contrast between air, soft tissue and bone was observed with a window width (WW) of 4013 and a window level (WL) of 653 units. The corresponding window settings for MRI were 1800 (WW) and 850 (WL). For a perfect match of MRI and CBCT, an appropriate zoom factor was chosen for each modality.

Anatomical structures were designated according to pertinent textbooks or publications [5,10,11,12]. 

## 3. Results

### 3.1. Cochlea

The cochlea can be depicted well on MRI as well as on CBCT. Basal, middle and apical turns are clearly shown. The osseous spiral lamina and the adjacent basilar membrane separate the scala vestibuli from the scala tympani. On CBCT, the osseous spiral lamina is visible as it originates from the modiolus. The basilar membrane, however, is visible only on MRI as it consists of soft tissue and contains no radiopaque calcific structures. The helicotrema is reliably pictured on MRI only. The scala media and the organ of Corti cannot be imaged in a direct way. Their position can only be inferred with the help of neighboring anatomical landmarks. The ductus reuniens is best shown on sagittal reformatted MRI.

### 3.2. Vestibule

CBCT clearly shows the osseous borders of the vestibule in all three standard planes. The depiction on MRI follows the osseous contours. The oval window including the stapes footplate is clearly recognizable on CBCT.

The topography of the saccule can only be deduced indirectly from the position of the inferior vestibular nerve and that of the utricle by the location of the superior vestibular nerve. The utricular and saccular maculae are shown on MRI in all three orthogonal standard planes. They appear as hypointense, dark areas within the membranous vestibulum.

### 3.3. Cochlear Aqueduct

The cochlear aqueduct containing the perilymphatic duct is seen on MRI as well as on CBCT. Especially on the axial slices, its origin from the basal turn can be delineated in the vicinity of the round window. The course of the cochlear canaliculus can be traced as far as its external aperture in the subarachnoid space.

### 3.4. Vestibular Aqueduct

The vestibular aqueduct includes the endolymphatic duct and is depicted by MRI and CBCT. Its typical course originating from the posterior border of the vestibule to the posterior aspect of the petrous pyramid is best shown on axial reformatted CBCT scans. The vestibular aqueduct terminates in the endolymphatic sac which is located in a dural duplicature, the position of which is identifiable on CBCT as a small dilation of the osseous canal. On MRI, the sac cannot be delineated. The course of the aqueduct is better depicted on CBCT than on MRI because the heavily T2-weighted MRI sequence can depict only the scarce aqueous fluid of the perilymphatic duct lumen but not the epithelium and stroma, whereas CBCT shows the complete inner osseous surface.

### 3.5. Semicircular Canals

The three semicircular canals are easily shown on CBCT and MRI and can be traced along their entire course. Their membranous compartment follows the contour of the bony border. However, it is not possible to differentiate the endolymphatic tube from the surrounding perilymph. The ampullae appear as subtle focal dilations of the canals, where on MRI, the ampullar crests are discerned as they protrude into the lumen of the ampullae as dark, hypointense spiny structures.

### 3.6. Internal Auditory Canal

The internal auditory canal with its orifice at the posterior aspect of the temporal bone is clearly seen on CBCT and MRI. The delineation of separate nerve bundles or the anterior inferior cerebellar artery (AICA) is not possible. This is partly due to the preparation of the specimen where the nerves had to be sacrificed, partly due to an air bubble which produced a susceptibility artifact on MRI. Likewise, the labyrinthine artery cannot be distinguished with certainty. In the lateral part of the internal auditory canal, the branches of the vestibulocochlear nerve are seen. The cochlear nerve is depicted with its anatomic relationship to the modiolus. The facial nerve is seen branching off above the cochlear nerve. The intermediate nerve is not visible. At the level of the outer geniculum of the facial nerve, an osseous dilation containing the geniculate ganglion can be seen. The entire course of the facial nerve can be traced in the temporal bone until its orifice at the stylomastoid foramen.

In the internal auditory canal, the vestibular nerve is seen on MRI dividing into the superior and inferior vestibular nerves as well as the posterior ampullar nerve. 

### 3.7. Tympanic Cavity

The tympanic cavity was not the focus of our study. Therefore, we describe our imaging findings only concisely.

CBCT is able to distinctly show the details of the whole ossicular chain including the stapes footplate at the oval window. The belly of the tensor tympani muscle is seen on CBCT and MRI, whereas its insertion at the manubrium mallei and its origin at the cochleariform process are visible only on CBCT. The position of the stapedius muscle can be inferred on CBCT by the position of the pyramidal eminence. The branching of the Chorda tympani within the tympanic cavity is visible on CBCT, whereas its further course cannot be delineated with certainty.

### 3.8. Comparison of CBCT and MRI

**Figure 1:**
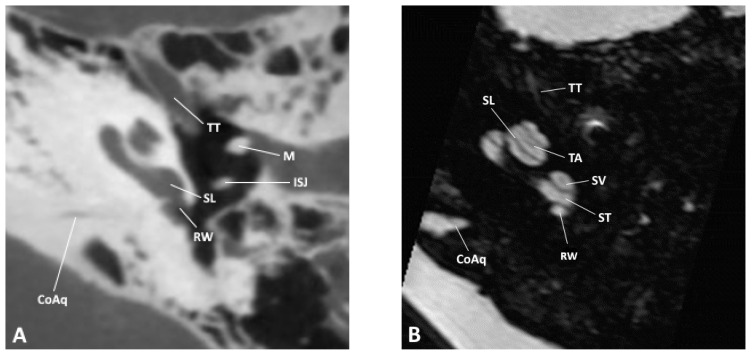
Axial slice through the inner and middle ear at the level of the round window as observed by (**A**) CBCT and (**B**) MRI; CoAq, cochlear aqueduct; ISJ, incudostapedial joint; M, malleus; RW, round window; SL, spiral lamina; ST, scala tympani; SV, scala vestibuli; TA, truncation artifact; TT, tensor tympani.

**Figure 2:**
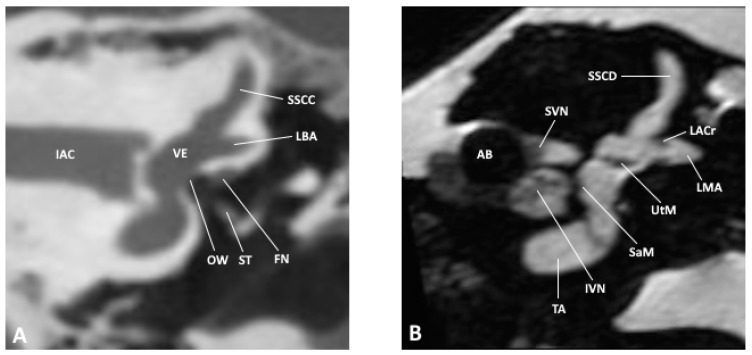
Coronal slice through the inner and middle ear at the level of the internal auditory meatus as observed by (**A**) CBCT and (**B**) MRI; AB, air bubble; FN, facial nerve; IAC, inner auditory canal; IVN, inferior vestibular nerve; LACr, lateral ampullary crest; LBA, lateral bony ampulla; LMA, lateral membranous ampulla; OW, oval window; SaM, saccular macula; SSCC, superior semicircular canal; SSCD, superior semicircular duct; ST, stapes; SVN, superior vestibular nerve; TA, truncation artifact; UtM, utricular macula; VE, vestibule.

**Figure 3:**
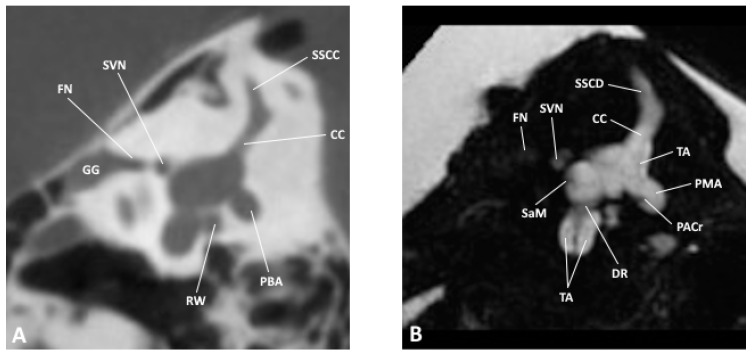
Sagittal slice through the inner and middle ear at the level of the vestibule as observed by (**A**) CBCT and (**B**) MRI; CC, common crus; DR, ductus reuniens; FN, facial nerve; GG, geniculate ganglion; PACr, posterior ampullary crest; PBA, posterior bony ampulla; PMA, posterior membranous ampulla; RW, round window; SaM, saccular macula; SSCC, superior semicircular canal; SSCD, superior semicircular duct; SVN, superior vestibular nerve.

**Figure 4:**
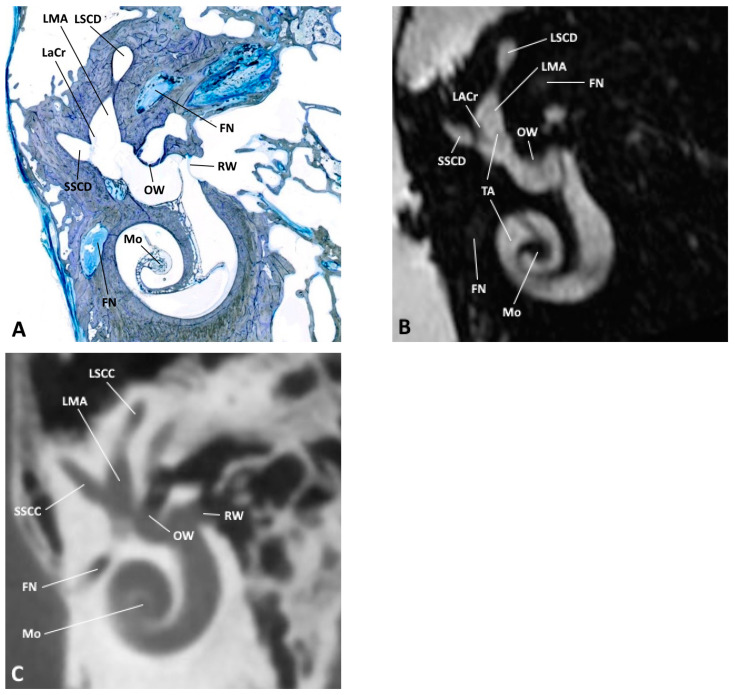
(**A**) Oblique anatomical section with the corresponding (**B**) MRI and (**C**) CBCT images; FN, facial nerve; LACr, lateral ampullary crest; LMA, lateral membranous ampulla; LSCC, lateral semicircular canal; LSCD, lateral semicircular duct; Mo, modiolus; OW, oval window; RW, round window; SSCC, superior semicircular canal; SSCD, superior semicircular duct; TA, truncation artifact.

Oblique anatomical whole-mount sections were regarded as the gold standard and were used as a reference whenever available. Figure 1, Figure 2, Figure 3 and Figure 4 show representative multiplanar MRI and CBCT scans and a comparison of imaging with the anatomy. A collation of the visibility of important anatomical structures is given in Table 1.

### 3.9. Artifacts

On MRI, an air bubble which was caught in the fundus of the internal auditory canal produced susceptibility artifacts which obscured the neurovascular bundle. When the CBCT scan was recorded at a later time, this bubble had meanwhile been absorbed.

Some linear hypointense structures traversing the bright signal of the inner ear on MRI had no correlate either on the histological whole-mount sections or in the relevant textbooks [12,13]. These structures mimic the delicate membranous border that separates the endolymphatic sac from the surrounding perilymphatic space. They run parallel to the bright/dark interface between bone and the aqueous contents of the inner ear at a certain distance defined by geometric imaging and the image reconstruction parameters. Therefore, we interpreted these linear structures as truncation artifacts. Examples are thin artifact lines in the cochlear scalae, in the semicircular canals or in the vestibulum where its outer borders are accentuated.

## 4. Discussion

Differentiation of anatomical details in the inner ear is a big challenge in modern cross-sectional imaging. Technical progress has allowed for the detailed delineation of the smallest anatomical details. This increases not only our understanding of physiological and pathological processes but also distinctly aids in the planning and execution of surgery.

The clinical introduction of inner ear imaging with a spatial and contrast resolution hitherto unknown opens a new understanding of pathology for which there is no tangible anatomical correlate. Knowledge of pathology presupposes familiarity with normal morphology. Radiological research on this topic is rare and the results of anatomical research are difficult to transfer to the mainly two-dimensional world of cross-sectional radiological imaging. It was the aim of this study to fathom the border of imaging, to name anatomical structures and to identify artifacts. Selecting anatomical whole-mount sections and microscopic anatomical monography [13] served as a worth reference.

Heavily T2-weighted 3D gradient echo sequences have stood the test of time in inner ear imaging. These techniques produce high-resolution isotropic images with a good SNR [4,14,15]. Their extremely high contrast between fluid and solid tissues is somehow counterbalanced by their comparatively poor contrast between individual kinds of tissue. However, the latter fact was not a disadvantage in our study as no soft tissue characteristics were considered.

It was the aim of the present study to depict the membranous inner ear with the maximum image resolution that a commercially available clinical 3T MRI scanner was able to produce when we started our examination. In order to avoid motion artifacts which are inevitable in living subjects, especially when long scanning times are necessary, we chose to perform our study on a fresh human ex vivo temporal bone specimen embedded in a physiologic saline solution. Previous scans with a formalin-fixated temporal bone had proven to be unsuccessful, which is in accordance with research reports [16]. 

To optimize the SNR, we chose a dedicated surface coil in the center of which the specimen was placed. Due to its transmit–receive (T/R) construction and digital signal transmission, this coil was able to combine a good SNR with the desired small FOV.

With our technique, we were able to depict the inner ear in excellent quality. The cochlea, vestibulum and semicircular canals could be visualized as well as the cochlear and vestibular aqueducts. Likewise, the utricular and saccular maculae and the ampullar crests of the semicircular canals were clearly visible. We succeeded in differentiating the nerves of the internal auditory canal, with their distal branching into the facial, cochlear, superior and inferior vestibular, as well as the posterior ampullary nerves. Contrary to clinical imaging of the internal auditory canal, where the elements of the neurovascular bundle are readily displayed [6], MR imaging in our specimen was degraded by a susceptibility artifact caused by an air bubble.

Of the membranous labyrinth, direct distinction between the peri- and endolymphatic compartment was not possible in our study, neither did we succeed in showing the scala media. This is in accordance with observations by Banciu [17] who studied the inner ear using a strongly T2-weighted 3D and fluid-attenuated inversion recovery (FLAIR) MRI technique with a comparable 3-Tesla scanner.

Van der Jagt [18] compared the visibility of inner ear structures between a 3T and a 7T scanner in patients with sensorineural hearing loss. At 7T, the better spatial resolution and the higher signal-to-noise ratio resulted in a more detailed presentation. The gain in anatomical information manifested itself, among others, in better visibility of the neural bundles within the inner auditory canal where even the intermediate nerve could be delineated. Out of the 26 inner ears depicted at 7T, the scala media could be shown in 7 cases in the first cochlear turn, and even in 21 cases in the second turn. At 3T, the scala media was not visible at all. The authors do not refer to the macular organs, the cochlear or vestibular aqueduct, nor to the endo- and perilymphatic ducts. As in our study, imaging artifacts deserve mentioning. At 7T, streak artifacts, especially at the cochlea, made the distinction of the scalae more difficult. These artifacts were not seen at 3T 

Van Egmont et al. also examined the inner ear anatomy [19,20]. On the one hand, they compared in vivo and ex vivo scans at 7T; on the other hand, they carried out a comparison of the image quality and visibility of inner ear structures in healthy volunteers between a 3T and a 7T scanner. They found out that all structures visible at 3T were seen at 7T with a better resolution. However, they were unable to image the scala media even at the highest resolution that was technically possible. The authors also faced problems with artifacts in high-resolution scans due to the location of the inner ear. One of the main reasons for this observation is the contrast between bony and air-filled components of the inner ear with fluid-filled compartments [21].

Proneness to susceptibility artifacts on MRI is more pronounced at a high magnetic flux density (“field strength”). These artifacts are unseen on CT or CBCT and must not be confused with true anatomical structures. They are due to the local inhomogeneity of magnetic fields in media of different magnetic properties, especially interfaces of soft tissue, bone and air [22]. On the images, susceptibility artifacts appear as geometric distortions and focal signal loss (dark areas), as was observed by us in the internal auditory meatus. We did not expect other sources of susceptibility artifacts, except some potential metallic abrasion along the cut edge of the specimen. However, we made no corresponding observations. 

Among clinically relevant MRI artifacts, truncation artifacts (also known as Gibb´s artifacts) deserve mentioning. Truncation artifacts appear as parallel lines oscillating along interfaces with high signal contrast, e.g., bone/fluid [23]. Truncation artifacts are the direct consequence of a Fourier transformation used to process the signals in MRI. Using this transformation, any graph can be approximated by a series of sine and cosine functions with a certain amplitude and frequency. Parallel to interfaces with a high and sudden jump in the signal, there is an oscillating overshoot at the high-signal side with a corresponding undershoot on the low-signal side. On an MRI scan, this artifact appears as bright and dark lines running parallel to high-contrast interfaces [24,25,26]. Truncation artifacts can be minimized by using a high imaging matrix and a small FOV, but technical limits cannot be eliminated completely as the scanning parameters cannot be adjusted ad libitum. In our study, with an extremely high inherent image contrast and a high geometric resolution, truncation artifacts can be the source of misinterpretation, which we attempted to avoid by visually referring to anatomical whole-mount sections.

It is a peculiarity of the T2-weighted imaging sequence we used that the bright signal of the fluid-filled compartments of the inner ear is immediately adjacent to the dark signal void of the surrounding bone. The truncation effect reaches its maximum at a distance of 4 pixels away from the signal jump [27]. This is in good accordance with our observation that the artifact was running about 1 to 2 mm parallel to the interface. 

On the MRI scans, we observed dark lines within the membranous labyrinth which could be confirmed neither at the whole-mount sections nor at another similar anatomical reference [28]. This, and the course of these lines at some distance and parallel to areas with signal jumps, confirmed our interpretation as truncation artifacts. In our study, we could observe these artifacts in the coils of the cochlea, especially the scala vestibuli and tympani, as well as in the vestibule and the semicircular canals.

Several authors have tested the hypothesis that an increase in flux density of the magnetic field (“field strength”) may lead to a better depiction of the delicate inner ear structures. The first examination of the inner ear using a 9.4-Tesla scanner was performed by Silver et al. in 2002 [29]. Before scanning, their temporal bone specimens were put in a saline solution containing the paramagnetic contrast medium Gadolinium DTPA at a concentration of 0.1 mmol/L. Their study yielded a detailed presentation of the cochlea, vestibular and semicircular canals. In addition to that, the vestibular membrane (Reissner´s membrane), the basilar membrane, the spiral ligament and, consequently, the scala media could be delineated.

Lane et al. [15] studied the anatomy of the membranous labyrinth of healthy volunteers at 3 Tesla. T2-weighted scans of a temporal bone scanned at 9.4 Tesla served as a reference. In analogy to our examination, at 3 Tesla, separation of the endolymphatic and perilymphatic compartments was not feasible without the use of an additional contrast medium, despite the highest technically possible resolution. Imaging of the utricular and saccular maculae succeeded already at 3 Tesla and could be confirmed at the 9.4-Tesla scan. Clear imaging of the scala media in the axial and sagittal slice orientation at the level of the basal and middle turn was another advantage of the stronger magnetic field.

Tylur et al. [21] investigated the anatomy of the inner ear of a temporal bone specimen at 11.7 Tesla. A cubic voxel size of 50 µm resulted in an extremely high spatial resolution. In the cochlea, a distinct subdivision into scala vestibuli, tympani and media was possible. The scala media could be delineated through imaging of the spiral ligament, the vestibular membrane and the basilar membrane. However, it was not possible to show further details of the basilar membrane including the organ of Corti. The individual neural bundles were readily displayed. Compared with our work, the ductus reuniens was more visible. The depiction of the utriculosaccular duct was not possible despite the high spatial resolution. In the vestibulum, the utricular and saccular maculae were clearly visible, as were the ampullar crests.

Imaging of the inner ear anatomy, especially differentiation of the endo- and perilymphatic compartments, is not only a challenge with regard to spatial resolution. A different approach is the use of paramagnetic contrast medium that is routinely used in clinical practice. After intra-tympanic application, the contrast medium is observed leaking into the perilymph, but not into the endolymph, making the imaging diagnosis of endolymphatic hydrops possible. Drawbacks of this method are its invasiveness, inconsistency of contrast enhancement and missing enhancement in the contralateral ear [30]. For this reason, in clinical routine, a less invasive procedure has taken root: after intra-venous application of the contrast medium, a delayed MRI scan using a specially designed FLAIR sequence shows enhancement of the perilymphatic space. One advantage of this method, apart from its lesser invasiveness, is the simultaneous enhancement of the perilymph on both sides, enabling, e.g., the diagnosis of bilateral endolymphatic hydrops [31,32].

Knowledge about the osseous anatomy and pathology of the temporal bone is often essential for diagnosis and for planning ear surgery. The depiction of osseous structures is inherent to all X-ray-based methods. Whereas conventional radiographs serve only for orientation or documentation, cross-sectional imaging (CT, CBCT) is the gold standard. Regarding temporal bone imaging, CBCT has some advantages over conventional multislice CT: slightly better spatial resolution, less radiation exposure and less susceptibility to beam-hardening artifacts (e.g., caused by metal) [33]. Hence, with regard to the laterobasis, CBCT is considered at least equivalent with CT [34,35].

Gupta et al. [36] compared multislice CT with CBCT in four temporal bone specimens. They found CBCT to be superior by virtue of its higher spatial resolution and less partial volume effects which resulted in an overall better image quality, especially when it came to the imaging of small bony structures. In a similar direct comparison of CT with CBCT in temporal bone specimens, Teymoortash et al. came to the same results [37]. In a retrospective image analysis of more than 200 temporal bone examinations, Güldner Ch et al. confirmed the benefit of CBCT in the diagnosis of middle ear and laterobasal pathologies and considered it at least equivalent with multislice CT [38]. Especially in the middle ear, the advantages of CBCT become evident where interruptions of the ossicular chain can be detected as well as subtle changes in the oval window in patients with otosclerosis [39]. Contrary to MRI, even the most advanced X-ray imaging modalities fail to depict the partially calcific structures of the utricular or saccular maculae within the confines of the inner ear.

At this point, synchrotron radiation X-ray microtomography (SRµCT) and Photon-Counting Detector CT (PCD-CT) should be outlined as experimental imaging methods for the visualization of delicate inner ear structures. Using SRµCT and its high resolution, Sismono et al. [40] were able to examine the finest inner ear structures, including Reissner’s membrane. New Photon-Counting Detector CT scanners are able to achieve improved quality in imaging the petrous bone through higher resolution with a smaller slice thickness [41]. Using synchrotron phase-contrast imaging, Mei et al. [42] succeeded in visualizing even the delicate vascular supply of the spiral ganglion. Although these methods are not established in clinical practice, they have the clear potential for future investigations.

In our study, the role of CBCT was mainly to facilitate the recognition of anatomical landmarks for MRI in the same specimen under investigation. The image quality and recognition of anatomical details correspond with research reports. Interestingly, all anatomical structures that were visible on CBCT could also be seen on MRI, whereas some elements of the inner structure of the endolymphatic and perilymphatic spaces were seen on MRI, but not on CBCT.

Previous studies have addressed MRI artifacts [7] and correlated MRI findings with histopathology [8]. However, imaging was performed on volunteers using clinical whole-head hardware, resulting in a limited spatial resolution. Therefore, their results cannot be transferred to our study. Signal loss due to partial volume averaging as observed by others is a relevant source of hypointensities in clinical settings. We minimized this confounder by using a geometric resolution as high as possible. In comparison to clinical whole-head imaging, our experimental setup and measurement design precluded aliasing (“fold-over” or “wrap-around”) artifacts that in clinical practice can be the source of significant misinterpretations. Likewise, chemical shift artifacts did not occur due to the scarcity of lipid in or near the inner ear and due to the low sensitivity of the measurement sequence chosen with regard to fat, neither did we observe any banding artifacts which can be misleading in T2*-weighted imaging sequences [7]. 

Our study was performed on a specimen with no known malformation of the head. It would be up to further research to correlate potential pathology of inner ear structures with deviations or dysplasia of the cranium as a whole.

One potential shortcoming of our investigation lies in the fact that we studied only one temporal bone specimen. However, this reflects only the formulation of our initial question: to distinguish true from artifactual structures as observed in clinical imaging. Our study successfully serves as a reference to denominate anatomy in everyday practice.

## 5. Summary

In the present study, we examined a human temporal bone specimen using clinically available high-definition MRI and CBCT.

To this end, a fresh temporal bone specimen was put in a physiologic saline solution and scanned in a 3-Tesla machine using a dedicated surface coil. In the second step, a CBCT scan of the same specimen was performed. After that, histological whole-mount sections were prepared and stained. Using multiplanar image reformatting techniques, we identified and labeled the relevant anatomical structures and compared image anatomy with histology.

In defining the outlines of the inner ear, CBCT and MRI were equivalent. MRI was clearly superior in depicting internal elements of the membranous labyrinth. However, the thin, membranous confines of the endolymphatic sac remained invisible. Truncation artifacts on MRI have to be distinguished from true anatomical structures. A discrimination of endolymphatic vs. perilymphatic compartment was not possible with either of the two imaging modalities and calls for different advanced imaging techniques. 

In conclusion, contemporary clinical cross-sectional imaging is capable of depicting delicate anatomical details of the inner ear. Our results could therefore serve as a reference for image analysis.

## Figures and Tables

**Table 1:** Visibility of selected anatomical structures, as observed on CBCT vs. MRI (+ visible, − not visible).

		CBCT			MRI	
	Axial	Coronal	Sagittal	Axial	Coronal	Sagittal
Spiral lamina	+	+	+	+	+	+
Utricular macula	−	−	−	+	+	+
Saccular macula	−	−	−	+	+	+
Oval window	+	+	+	+	+	+
Round window	+	+	+	+	+	+
Superior ampullar crest	−	−	−	+	+	+
Lateral ampullar crest	−	−	−	+	+	+
Posterior ampullar crest	−	−	−	+	+	+
Cochlear nerve	+	+	+	+	+	+
Super vestibular nerve	+	+	+	+	+	+
Inferior vestibular nerve	+	+	+	+	+	+
Posterior vestibular nerve	+	+	+	+	+	+
Cochlear aqueduct	+	+	+	+	+	+
Vestibular aqueduct	+	+	+	+	+	+
Ductus reuniens	−	+	+	−	+	+

## Data Availability

A complete set of images is available from the author upon request.
